# Defining phylogenetic relationship of *Nepeta* x *tmolea* and its parents via DNA barcoding

**DOI:** 10.3897/phytokeys.134.38238

**Published:** 2019-10-23

**Authors:** Taner Özcan

**Affiliations:** 1 Balıkesir University, Faculty of Necatibey Education, Department of Biology Education Balıkesir, Turkey Balıkesir University Balıkesir Turkey

**Keywords:** hybrid, molecular, *
Nepeta
*, phylogeny, Turkey

## Abstract

*Nepeta viscida* and N. nuda subsp. nuda and N. × tmolea were examined in this study. Mainly fresh leaf pieces, dried with silica grains, were used for DNA extraction procedures via DNA isolation kits. Standard PCR techniques were executed using three different primer sets (one nuclear DNA region (nrITS) and two chloroplast DNA regions (*rpl*32-*trn*L and *trn*A(Leu)-*trn*A(Phe)-*trn*L-F). DNA sequences were analysed and evaluated using different molecular approaches and software. Consequently, the inconstant molecular structure and hybrid nature of N. × tmolea specimens were shown and interpreted in this study. According to our result, N. × tmolea have some intermediate characters compared to its parents. nrITS data give more information phylogenetically, and also the most polymorphic loci are seen in nrITS data. Morphological and molecular data contribute to define separation of N. × tmolea. Consequently, the inconstant molecular structure and hybrid nature of N. × tmolea specimens were shown and interpreted in this study.

## Introduction

Lamiaceae family – the mint family – members are well known for their medicinal and aromatic properties in the pharmaceutical industry. The *Nepeta* L. genus is mainly native to Europe, Western Siberia, Far East and North Asia and consists of approximately 300 species with its being one of the largest genera in Lamiaceae (Pojarkova 1954; Hedge 1986; Jamzad et al. 2000, 2003b; Tzakou et al. 2000; Mojab et al. 2009). In recent studies, Turkish *Nepeta* members have been represented by 44 species. Twenty-two of these species are endemic to Turkey (Aytaç and Yıldız 1996; Güner et al. 2000; Dirmenci 2003) with the distribution areas of the species being mainly in east Anatolia and the Taurus Mountains in Turkey (Dirmenci 2005). Nepeta nuda L. subsp. nuda is a widespread and well-known subspecies of *N. nuda* in Turkey with its distinguishing characters of violet-blue calyx and corolla (Hedge and Lamond 1982; Dirmenci 2003). Nepeta nuda subsp. nuda and *N. viscida* Boiss. are members of Group A, according to the Flora of Turkey classification (Hedge and Lamond 1982; Dirmenci 2003). *Nepeta viscida* is readily separated from N. nuda subsp. nuda by its viscous glandular trichomes and general habit.

It is mentioned in the Flora of Turkey that *N. viscida* hybridises with *N. nuda* in overlapping areas and forms the hybrid described as N. × tmolea Boiss. (Hedge and Lamond 1982). In the field trips during this study, we found some N. nuda subsp. nuda and *N. viscida* individuals that reflect their typical characters. Some individuals had, however, some intermediate morphological characters: they were not viscid and their stem, leaf and corolla colours were quite different from N. nuda subsp. nuda and *N. viscida*. Thus, we recognised these specimens as N. × tmolea. Some N. × tmolea hybrid individuals were more similar to *N. viscida* in terms of general habits, calyx and leaf characters; on the other hand, some samples were more similar to N. nuda subsp. nuda in terms of their bluish colour on the verticillasters and their having no adhesive glandular trichomes.

According to literature, trichome types, density, presence/absence etc. are very important characters for identifying different taxa in the Lamiaceae family (Husain et al. 1990; Ecevit-Genç et al. 2015, 2017, 2018; Krawczyk and Głowacka 2015; Sajna and Sunojkumar 2018) and, of course, the genus *Nepeta* (Kolalite 1988; Dirmenci 2003; 2005; Kaya et al. 2007; Açar et al. 2011; Yarmoohammadi et al. 2017; Özcan 2019). Additionally, it is mentioned in the studies that, although the type and density of trichomes are distinctive amongst species, they can vary in different organs of the same individual.

DNA barcoding methods have been frequently used in differentiating taxa in recent years (Hebert et al. 2003). Specimens can be separated by obtaining a standard DNA region using a very small sample (Kress and Erickson 2007). According to Jamzad et al. (2003a), nuclear ITS DNA sequences are correlated with some morphological characters and, thus, this region can be helpful in defining the phylogenetic positions of the *Nepeta* species. Molecular approaches are also used to reveal heterozygotic and polymorphic structures of some hybrid taxa belonging to the Lamiaceae family in literature (Bariotakis et al. 2016; Kokubugata et al. 2011; Jedrzejczyk 2018; Dirmenci et al. 2018a, 2018b, 2019a). Some Single Nucleotide Polymorphisms (SNPs), which are are the most common type of genetic variation among plants and meaning replacing of a nucleotide (i.e. C) to another (i.e.T) in a certain stretch of DNA, were identified in this study.

This research aimed to reveal the phylogenetic relationships and heterozygous DNA structure of Nepeta nuda subsp. nuda, *N. viscida* and their hybrid N. × tmolea. The internal transcribed spacers of nuclear ribosomal DNA (nrITS), *trn*L-F and *rpl*32 regions from chloroplast DNA were examined to define heterozigoty of DNA sequences amongst parents and hybrid specimens.

## Materials and methods

### Plant materials

**Figure 1.**
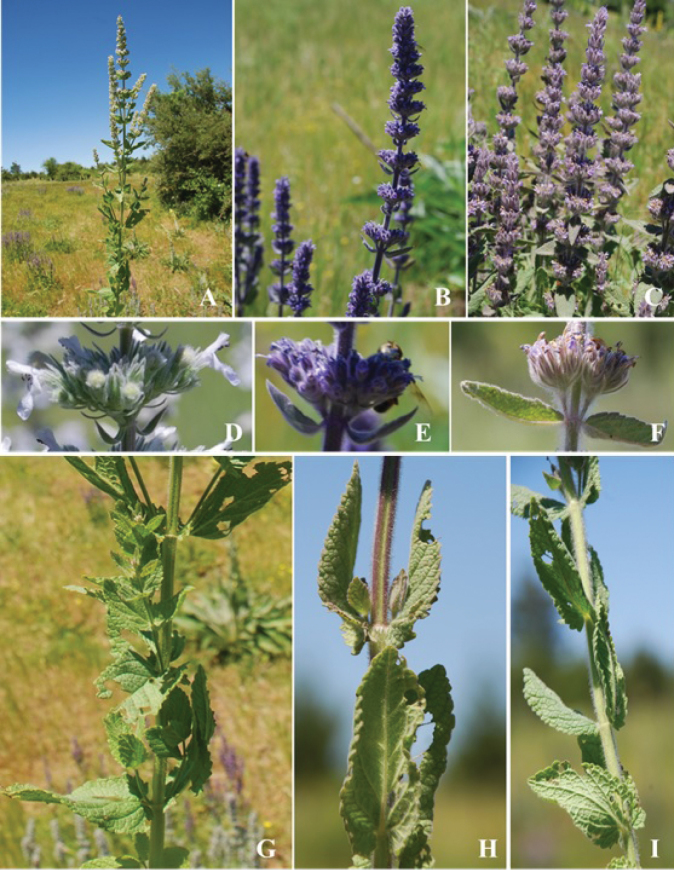
General habit, inflorescence and lower parts of N. nuda subsp. nuda (**A, D, G**); N. × tmolea (**B, E, H**) and *N. viscida* (**C, F, I**).

The different individuals of *N. viscida*, N. × tmolea and N. nuda subsp. nuda were collected during the field trips (2016–2018) from their natural habitats in Balıkesir (Dursunbey-Çamlık) (Fig. 1), İzmir (Ödemiş-Bozdağ) and from Kütahya in 2002. Voucher specimens are deposited in the Herbarium of Necatibey Education Faculty of Balıkesir University in Balıkesir, Turkey.

### DNA isolations

DNA isolations were performed using the DNeasy Plant Mini Kit (QIAGEN, Germany), following the manufacturer’s instructions with some modifications. Eight different N. × tmolea specimens and different specimens of *N. viscida* and N. nuda subsp. nuda were used for DNA isolations. Taxon name, voucher number and localities are given in Table 1.

**Table 1.** Voucher information of *Nepeta* species examined for DNA extractions.

Taxon	Voucher number	Locality
*N. viscida*	4759	**Balıkesir**: Dursunbey, Alaçam Mount., Gölcük, around Karaveli Hill, 39.42650N, 28.53057E, 4970 ft alt., 19/06/2017.
	4762	**Balıkesir**: Dursunbey, Alaçam Mount., Sokuldak area, 39.43673N, 28.51373E, 4790 ft alt., 19/06/2017.
	4766	**İzmir**: Ödemiş, around Bozdağ ski resort, 20/06/2017.
	4768	**İzmir**: Ödemiş, around Bozdağ ski resort, 20/06/2017.
	5024	**Balıkesir**: Dursunbey, Alaçam Mount., around Karaveli Hill, 39.42625N, 28.53123E, 4930 ft alt., 11/06/2018.
	5027	**Balıkesir**: Dursunbey, Alaçam Mount., around Karaveli Hill, 39.42682N, 28.52975E, 4960 ft alt., 11/06/2018.
	5030	**Balıkesir**: Dursunbey, Alaçam Mount., Sokuldak area, 39.43662N, 28.51364E, 4780 ft alt., 11/06/2018.
N. × tmolea	4758	**Balıkesir**: Dursunbey, Alaçam Mount., Gölcük, around Karaveli Hill, 39.42650N, 28.53057E, 4970 ft alt., 19/06/2017.
	4761	**Balıkesir**: Dursunbey, Alaçam Mount., Sokuldak area, 39.43673N, 28.51373E, 4790 ft alt., 19/06/2017.
	4765	**İzmir**: Ödemiş, around Bozdağ ski resort, 20/06/2017.
	4770	**İzmir**: Ödemiş, around Bozdağ ski resort, 20/06/2017.
	5023	**Balıkesir**: Dursunbey, Alaçam Mount., around Karaveli Hill, 39.42625N, 28.53123E, 4930 ft alt., 11/06/2018.
	5026	**Balıkesir**: Dursunbey, Alaçam Mount., around Karaveli Hill, 39.42682N, 28.52975E, 4960 ft, 11/06/2018.
	5029	**Balıkesir**: Dursunbey, Alaçam Mount., Sokuldak area, 39.43662N, 28.51364E, 4780 ft alt., 11/06/2018.
	1073	**Balıkesir**: Dursunbey, above Tahtalık Hill, 5413 ft alt., 07/05/2000.
N. nuda subsp. nuda	4757	**Balıkesir**: Dursunbey, Alaçam Mount., Gölcük, around Karaveli Hill, 39.42650N, 28.53057E, 4970 ft alt., 19/06/2017.
	4764	**İzmir**: Ödemiş, around Bozdağ ski resort, 20/06/2017.
	4769	**İzmir**: Ödemiş, around Bozdağ ski resort, 20/06/2017.
	5021	**Balıkesir**: Dursunbey, Alaçam Mount., Soğucak area, 39.45649N, 28.53786E, 3818 ft alt., 11/06/2018.
	5022	**Balıkesir**: Dursunbey, Alaçam Mount., around Karaveli Hill, 39.42625N, 28.53123E, 4930 ft alt., 11/06/2018.
	5025	**Balıkesir**: Dursunbey, Alaçam Mount., around Karaveli Hill, 39.42682N, 28.52975E, 4960 ft alt., 11/06/2018.
	5028	**Balıkesir**: Dursunbey, Alaçam Mount., Sokuldak area, 39.43662N, 28.51364E, 4780 ft alt., 11/06/2018.
	1940	**Kütahya**: Radar road, 3935–4920 ft alt., 07/10/2002.

### PCR amplification

In this study, molecular analyses of N. × tmolea, *N. viscida* and N. nuda subsp. nuda were carried out using three different DNA regions: the nuclear internal transcribed spacer (nrITS), trnA (Leu)-trnA (Phe) (trnL-F) and rpl32-trnL regions of the chloroplast DNA (cpDNA). PCR amplification of the ITS nrDNA were performed using ITS5a (5'-CCT TAT CAT TTA GAG GAA GGA G-3') (Stanford et al. 2000) and ITS4 (5'-TCC TCC GCT TAT TGA TAT GC-3') (White et al. 1990) primers. The rpl32- trnL cpDNA amplifications were performed using rpl32-F (5'-CAG TTC CAA AAA AAC GTA CTT C-3') (Shaw et al. 2007) and trnL (UAG) (5'-CTG CTT CCT AAG AGC AGC GT-3') (Shaw et al. 2007) primers and the trnL-F amplifications were performed with trnL-c (5’-CGA AAT CGG TAG ACG CTA CG-3’) (Taberlet et al. 1991) and trnL-f (5’-ATT TGA ACT GGT GAC ACG AG-3’) (Taberlet et al. 1991) primers.

### DNA data analysis

The PCR products that were successfully amplified and checked on agarose gel were sent to Genoks (Gene Research and Biotechnology Company, Turkey) for sequencing. Raw sequenced DNA data files were edited via Sequencer version 5.4 (Gene Codes Corporation, Ann Arbor, MI, USA) and edited sequences were aligned using Bioedit 7.2.5 (Hall 1999). The polymorphic sequence loci and heterozygous structure of *N. viscida*, N. × tmolea and N. nuda subsp. nuda were identified and polymorphisms of these specimens were demonstrated by selected software. Successfully sequenced specimens were given in Table 1. Phylogenetic cladograms were constructed using PAUP* 4.0a165 (Swofford 2003) and Dendroscope (Huson and Scornavacca 2012), and a Neighbour-Net split graph was conducted using SplitsTree 4.14 (Huson and Bryant 2006). A data matrix was constructed according to discriminative characters belonging to *rpl*32 DNA data. In addition, discriminant analysis was carried out with PAleontoSTatistics (PAST) (Hammer et al. 2001) to show the position of individuals in these studies.

## Results

*N. viscida* is easily morphologically distinguished from N. nuda subsp. nuda by its sticky stem and leaves. These sticky structures, resulting from viscous glandular trichomes, are highly characteristic for *N. viscida* in *Nepeta* genus (Dirmenci 2003; Özcan 2019). Although this morphological feature is very significant, *N. viscida* and N. nuda subsp. nuda taxa are classified under Group A (Hedge and Lamond 1982; Dirmenci 2003). According to the Flora of Turkey, *N. viscida* and *N. nuda* belong to Group A along with *N. cataria* L., *N. isaurica* Boiss. & Heldr. ex Benth. and *N. caeserea* Boiss. Özcan (2019) stated that N. × tmolea is quite different from its parents but micromorphologically more similar to N. nuda subsp. nuda. The indumentum is a distinctive character for distinguishing *N. viscida* and N. nuda subsp. nuda. As with many hybrids (Clevinger and Panero 2000; Baumel et al. 2002; Lowe and Abbott 2004; Liu et al. 2017; Szczecińska et al. 2017; Jaźwa et al. 2018; Van Valkenburg et al. 2018; Dirmenci et al. 2019b), N. × tmolea hybrid individuals show transition/intermediate characteristics between *N. viscida* and N. nuda subsp. nuda. However, some specimens of N. × tmolea have higher similarities to N. nuda subsp. nuda, while others have high morphological similarities to *N. viscada*. Namely, N. × tmolea is distinguished from N. nuda subsp. nuda by its bracteoles 5–10 mm (not 2.5–4 mm), calyices 6–9 mm (not 3.5–4 mm), calyx teeth 3.5–5 mm (not 1.5–2 mm), corolla 7.5–10 mm (not 5.5–6.5 mm). Also, it differs from *N. viscida* by its bracteoles 5–10 mm (not 8.5–11 mm), calyices 6–9 mm (not 8.5–12 mm), calyx teeth 3–5 mm (not to 6.5 mm), and corolla 7.5–10 mm (not 9–13 mm).

We used three different loci, one nuclear DNA loci-nrITS- and two DNA loci from chloroplast genome-*rpl*32-*trn*L and *trn*A(Leu)-*trn*A(Phe)- in this study.

### Nuclear DNA data

**Figure 2.**
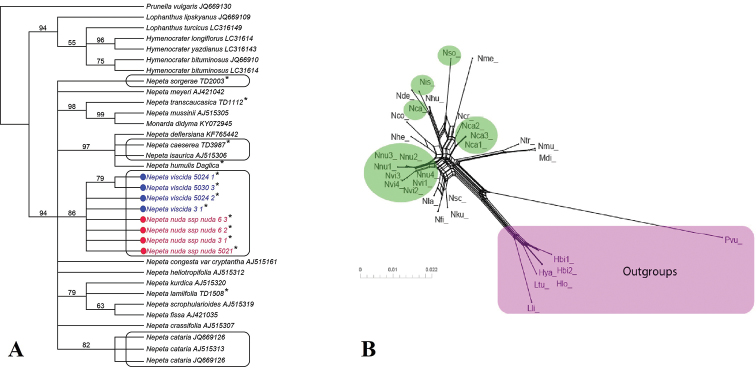
Phylogenetic position of *N. viscida* and N. nuda subsp. nuda amongst different *Nepeta* species and outgroups (based on nrITS sequences and Maximum Likelihood phylogram (**A**) and Neighbour-Net Diagram (**B**) without hybrids.(* examined taxa in this study).

In total, 21 taxa were sequenced for the ITS sequence matrix. In the parsimony heuristic search, consistency, retention and homoplasy indices were identified as 0.75, 0.78 and 0.25, respectively. According to Fig. 2, *N. viscida* and N. nuda subsp. nuda are sister taxa and belong to the same clade in comparison to other Group A members with a strong bootstrap value (86). When Fig. 1B is analysed, the *N. viscida*-N. nuda subsp. nuda group has a close relationship with *N. kurdica* Hausskn. & Bornm., *N. fissa* C.A.Mey, *N. scrophularioides* Rech.f. and *N. lamiifolia* Adam ex G.F.Hoffm. When the nrITS sequences of N. × tmolea and its parents are compared, N. × tmolea has 8 single nucleotide polymorphisms (Table 2). As mentioned above, N. × tmolea has some intermediate characters between its parents, such as leaf size and indumentum density, and our DNA data contribute further with the morphological characters. N. nuda subsp. nuda (1940) and N. nuda subsp. nuda (4764 and 4769) individuals (distributing in Ödemiş, see Table 1) differed the specimens from Balıkesir-Dursunbey (4757 and 5021). Thus, nrITS data also gave us intra-individual differentiations.

All the nrITS DNA data included 594 characters; 579 of 594 characters were constant, 6 variable characters were parsimony uninformative and 9 of the rest were parsimony informative (Table 2). *Nepeta viscida* 5024-4, 5024-2 and 5030-1 specimens have different nucleotides at the nucleotide positions of 11, 353, 420, and 462 in comparison to *N. viscida* 5024-1, 5030-3 and 5024-3 specimens, which are distributing in the same location (Dursunbey). In addition, the most heterozygous individual, *N. viscida* 5024-3 has heterozygote nucleotide polymorphisms at positions 355, 420 and 462. The most polymorphic locus is seen at position 421 (C-T nucleotide heterozygous - in bold characters) for all the specimens. On the other hand, all the examined taxa have polymorphic loci, according to nrITS data. These heterozygote sequences may be the result of continuous crossing between *N. viscida* and N. nuda subsp. nuda and backcrossing amongst the parents and N. × tmolea. Additionally, it can be seen from the Table 1 that, not only studied *N. viscida* members (7 specimens), but also N. nuda subsp. nuda (5 specimens) members have heterozygous structures, not only constant characters, at the given nucleotide positions.

**Table 2.** Separated loci of *N. viscida*, N. nuda subsp. nuda and N. × tmolea based on nrITS data.

	1	4	1	3	4	4	4	4	5
	1	3	2	5	2	2	6	7	3
			0	5	0	1	2	4	1
*Nepeta viscida* 5024 4	C	C	T	T	G	T	T	G	C
*Nepeta viscida* 5024 2	C	C	G	T	G	**C/T**	T	**G/T**	C
*Nepeta viscida* 5030 1	C	C	G	T	G	**C/T**	T	T	C
*Nepeta viscida* 5024 1	A	C	G	A	T	T	A	G	C
*Nepeta viscida* 5030 3	A	A	G	A	T	T	A	G	C
*Nepeta viscida* 5024 3	A	C	G	**A/T**	**G/T**	T	**A/T**	G	C
*Nepeta viscida* 4759	A	C	G	**A/T**	G	T	T	G	C
*Nepeta* × *tmolea_*4758	A	C	G	A	G	**C/T**	**A/T**	G	T
*Nepeta* × *tmolea_*5023 3	C	C	T	T	G	T	T	G	C
*Nepeta* × *tmolea_*5023 2	C	C	T	T	G	T	T	**G/T**	C
*Nepeta* × *tmolea_*4761	C	C	G	T	G	**C/T**	T	**G/T**	C
*Nepeta* × *tmolea_*1073	C	A	G	T	G	**C/T**	T	T	C
*Nepeta* × *tmolea_*5029 2	C	A	G	T	G	**C/T**	T	T	C
Nepeta nuda subsp. nuda 5021	C	C	T	T	G	T	T	G	C
Nepeta nuda subsp. nuda 4757	C	C	G	**A/T**	G	T	**A/T**	G	T
Nepeta nuda subsp. nuda 1940	C	C	G	**A/T**	G	T	**A/T**	G	T
Nepeta nuda subsp. nuda 4769	C	C	G	T	G	**C/T**	T	**G/T**	C
Nepeta nuda subsp. nuda 4764	C	A	G	**A/T**	G	**C/T**	T	**G/T**	C

**Figure 3.**
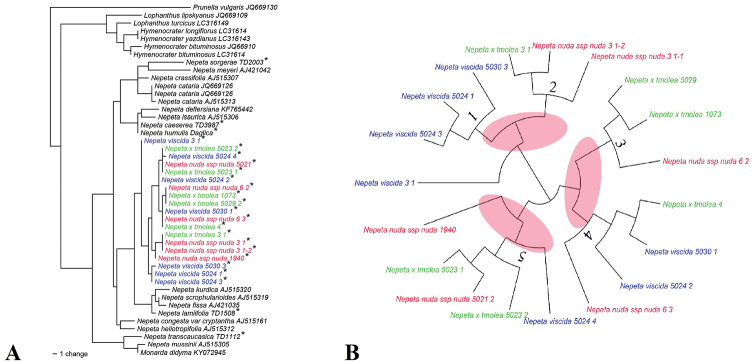
Phylogenetic relationship of *N. viscida*, N. nuda subsp. nuda and N. × tmolea with some *Nepeta* members and outgroups (based on nrITS sequences and Neighbour Joining phylogram (**A**) and Dendroscope diagram (**B**).(* examined taxa in this study).

According to nrITS sequences, different N. × tmolea specimens are classified with different parents (Fig. 3). Five main clades can be seen in Fig. 2. Two parents and their putative hybrid specimens share the same clade at clades 4 and 5, N. nuda subsp. nuda and N. × tmolea are more similar at clades 2 and 3. Therefore, it can be considered that the phylogenetic position of N. × tmolea is not constant and that ancestral species show transitions in different clades. When the hybrid individuals are not included in the phylogenetic analysis, *N. viscida* and N. nuda subsp. nuda tend to be closer to individuals of their own species, but ancestral species are divided into different clades after adding hybrid sequences in the analysis.

### Chloroplast DNA data

*rpl*32-*trn*L and *trn*L-F DNA regions were examined from the chloroplast genome. The longest data of studied regions were obtained from *rpl*32-*trn*L sequences. A total of 891 nucleotides were obtained from 29 specimens belonging to the parents and hybrid taxa; 855 of 891 characters were constant and parsimony-informative characters were 31. On the other hand, 847 characters were obtained from 32 specimens belonging to the parents and hybrid taxa, 833 of which were constant and 10 characters of the rest of the sequences were parsimony-informative for the *trn*L-F region.

**Figure 4.**
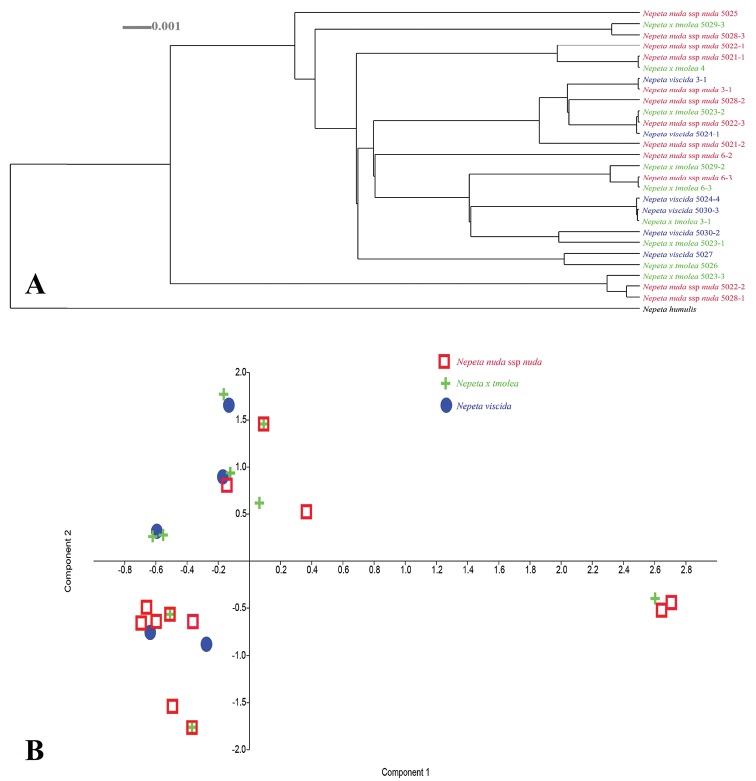
Dendroscope cladogram and PCA diagram based on *rpl*32-*trn*L data.

When we analyse Fig. 4, the phylogenetic tree and PCA diagram show us the transition amongst the species and hybrid individuals. This means that neither *N. viscida* nor N. nuda subsp. nuda specimens are monophyletic. Some clades have only one putative ancestor and hybrid and some of them have parents and hybrid taxa. These three taxa are mixed together and grouped at different clades in the cladogram (Fig. 4A) or at different regions in the PCA diagram (Fig. 4B). In addition, three N. × tmolea samples have completely similar DNA sequences with three N. nuda subsp. nuda samples and this can also be seen from the PCA diagram (with black arrows) (Fig. 4B).

**Figure 5.**
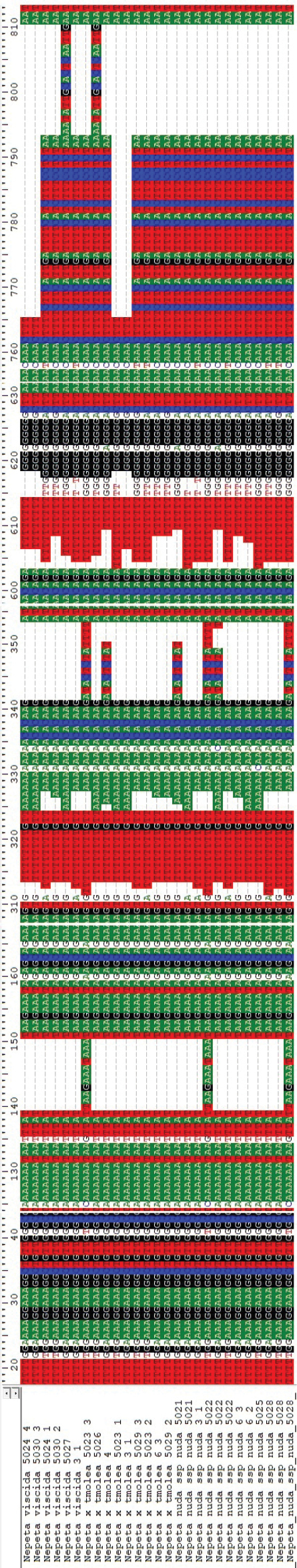
Insertions, deletions and single nucleotide polymorphisms based on *rpl*32-*trn*L sequences.

Single nucleotide polymorphisms (SNPs) were mostly seen in *rpl*32 data. G-T polymorphisms at positions 22, 41, 135, A-G polymorphisms at positions 24, 160, 311, A-C polymorphisms at positions 45, 331, 334 and C-T polymorphism at position 758 are significant for distinguishing specimens. Insertion-deletion sites are very significant, especially at the nucleotide positions between 140–150, 312–314, 325–328, 340–353, 603–608 and the longest one between positions 764–810 (Fig. 5).

**Figure 6.**
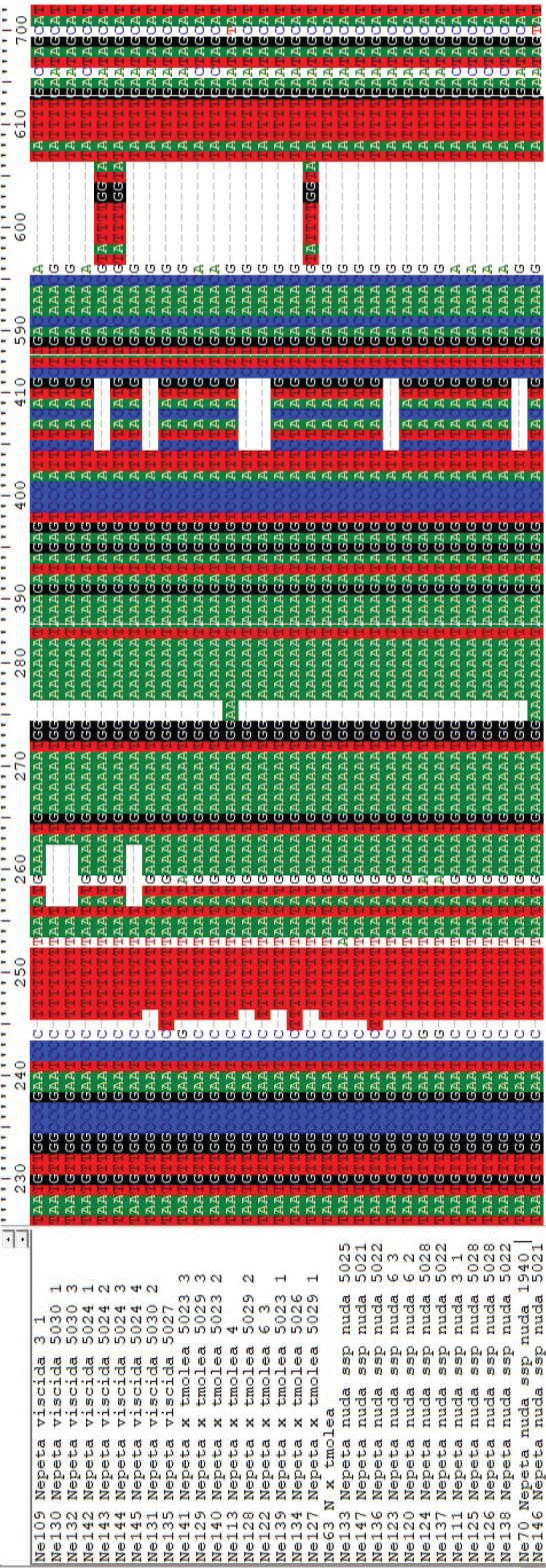
Insertions, deletions and single nucleotide polymorphisms based on *trn*L-F sequences.

*trn*L-F has also some SNPs at the nucleotide positions of 244, 596 and 696. Insertion-deletion (I-D) sites in *trn*L-F data are shorter than *rpl*32 data. There are three parsimony-informative I-D regions around the nucleotides 260, 410 and 600 (Fig. 6). Unfortunately, insertion or deletion sites were not parsimony informative for our finding out phylogenetic position of the species.

## Conclusions

Possible hybridisation between N. nuda subsp. nuda and *N. viscida* was estimated by Boissier (1859) for the first time but N. × tmolea was not presented as a hybrid. According to morphological studies, although general habitus, calyx and leaf characters of N. × tmolea are more similar to *N. viscida*, its indumentum (especially glandular trichome) is very different and separated. Molecular data overlaps with morphological data. As in the morphological data, hybrid individuals have intermediate characters in DNA sequences, and these characters occur as polymorphic loci.

DNA sequences, especially nrITS data, have been used by many scientists to discover the phylogenetic position and relationship of numerous species in literature. In this study, nrITS gave information about SNPs and *rpl*32-*trn*L and *trn*L-F were used to specify the parents’ taxa N. × tmolea. Having some polymorphic loci of N. nuda subsp. nuda (Table 2) has probably caused introgression. Hybrid forming areas (Dursunbey and Ödemiş districts) of N. nuda subsp. nuda and *N. viscida* are mostly contacted and formed N. × tmolea. In these hybrid swarm regions, N. × tmolea individuals possibly do backcrossing with its parents. Additionally, because of this backcrossing, some N. nuda subsp. nuda specimens have different nucleotides from the other N. nuda subsp. nuda samples which are the original parental individuals. According to literature, while chloroplast DNA gives us information about maternal or paternal inheritance, this study did not provide a completely reasonable result based on *rpl*32-*trn*L and *trn*L-F data.

In addition, we could not see logical clustering among the specimens growing in the same location (Dursunbey or Ödemiş), and nrITS data also gave us intra-individual differentiations of *N. viscida* and N. nuda subsp. nuda.
